# The tumor or inflammation? a case report on primary pulmonary choriocarcinoma

**DOI:** 10.3389/fonc.2023.1108798

**Published:** 2023-07-13

**Authors:** Na Wang, Nan Zhang, Xinyue Zhang, Yuanyuan Wang, Yajie Fu, Lingfei Guo, Changhu Liang, Mengru Yu

**Affiliations:** ^1^ Department of Radiology, Shandong Provincial Hospital Affiliated to Shandong First Medical University, Jinan, Shandong, China; ^2^ Department of Medical Imaging, Binzhou Medical University, Yantai, Shandong, China; ^3^ Department of Medical Ultrasound, The First Affiliated Hospital of Shandong First Medical University & Shandong Provincial Qianfoshan Hospital, Shandong Medicine and Health Key Laboratory of Abdominal Medical Imaging, Jinan, Shandong, China; ^4^ Shandong Key Laboratory of Reproductive Medicine, Department of Obstetrics and Gynecology, Shandong Provincial Hospital Affiliated to Shandong First Medical University, Jinan, Shandong, China

**Keywords:** primary pulmonary choriocarcinoma, β-human chorionic gonadotropin, differential diagnosis, computerized tomography, treatment

## Abstract

Choriocarcinoma is a rare malignant germ cell neoplasm with high invasiveness, the majority of which are pregnancy-related, and the female genital tract is the most prevalent site of the disease. Although early-stage choriocarcinoma typically metastasizes to the lungs, primary pulmonary choriocarcinoma is extremely rare. Primary pulmonary choriocarcinoma is difficult to diagnose, and it progresses rapidly. Combined with the difficulty of treatment, the prognosis of patients is generally poor. In this article, we retrospectively analyzed a case of female primary pulmonary choriocarcinoma, combined with a review of literature, to understand and describe the diagnostic and treatment progress of PPC.

## Introduction

Choriocarcinoma is a highly invasive malignant tumor originating from the trophoblast epithelium of pregnancy. In rare cases, choriocarcinoma can originate in the external genital region, predominantly in midline structures such as the retroperitoneum, mediastinum, and pineal gland (1, 2). Tumor tissue is composed of cytotrophoblasts, syncytiotrophoblasts, and variable intermediate trophoblasts that secrete β-human chorionic gonadotropin (β-hCG) (3, 4). Primary choriocarcinoma of the lung is very rare and presents mainly as hemoptysis with pulmonary nodules (5); it primarily affects young adults and has a poor prognosis, with a 5-year survival rate of less than 5% (2, 6). In clinical practice, due to the atypical symptoms of primary pulmonary choriocarcinoma (PPC), it is particularly easy to misdiagnose or delay diagnosis. Here, we report the diagnosis and treatment of primary pulmonary choriocarcinoma in a 27-year-old woman.

## Case presentation

**Figure 1:**
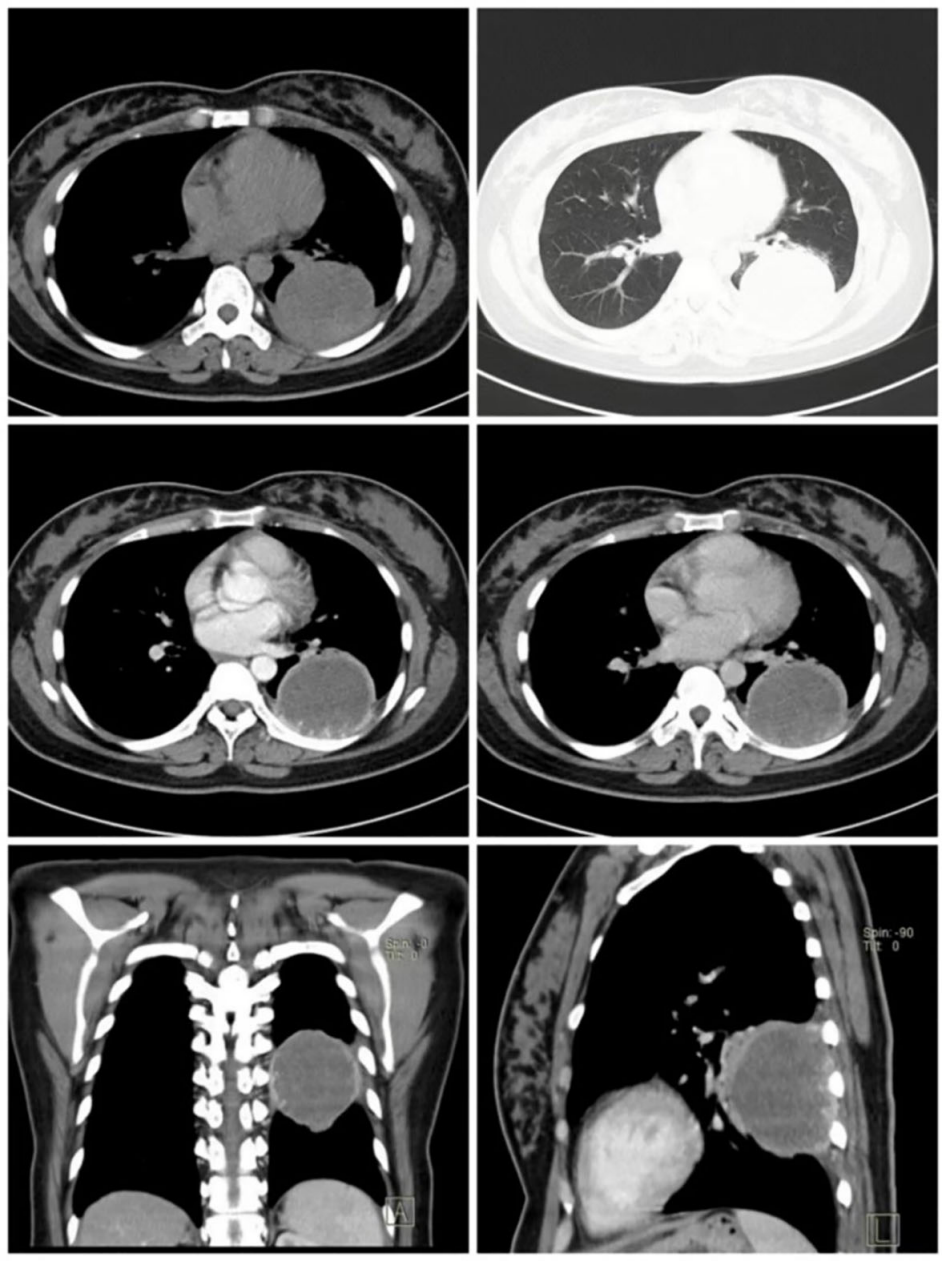
A chest CT scan revealed a large round lesion, approximately 6 cm in diameter, in the lower lobe of the left lung, with a well-defined border and homogeneous density. Following the enhancement scan, the edge of the lesion showed obvious ring enhancement, and villous tissue protruding inward.

A 27-year-old woman presented to the hospital with a cough, blood-stained sputum, and a fever for 1 month and chest pain for 10 days. The patient reported that she was married at an appropriate age, had regular menstruation, and had two pregnancies, 3.5 and 1.5 years before her admission to the hospital. There were no similar or hereditary disorders in her family. The physical examination, including the lungs, heart, peripheral lymph nodes, abdomen, and external anal genitalia, showed normal results, and no abnormalities were detected in other organs. A chest computerized tomography (CT) scan in a local hospital showed a space-occupied lesion in the lower lobe of the left lung. However, the anti-infective treatment was ineffective. A second chest CT scan showed an enlarged space occupying lesion in the lower lobe of the left lung (**Figure 1**), which was diagnosed as an infected-pulmonary cyst.

Preliminary laboratory examination revealed that the level of non-small cell lung cancer (NSCLC)-associated antigen was 6.62 ng/ml and the level of neuron-specific enolase was 16.73 ng/ml, indicating a malignant lung tumor.

At first, the patient was diagnosed with pulmonary cyst associated with infection according to the fever symptoms and CT examination results, but after a period of antibiotic treatment, there was no obvious effect. After the second CT examination, the lesion in the left lower lobe of the lung became larger than before in a short time, and the values of neuron-specific enolase and NSCLC-associated antigen were relatively high. Although it was still uncertain whether the lesion was inflammation or tumor, considering the high possibility of lung cancer, the decision was made to perform surgical resection of the lung lesion of the lower left lobe. The patient had surgical indications, and there was no evidence of other lesion in the preoperative imaging examinations, including emission computed tomography (ECT), brain magnetic resonance imaging (MRI), and abdominal ultrasound. Meanwhile, the cardiopulmonary risk of surgical resection was within the acceptable range. Therefore, video-assisted thoracoscopic surgery was proposed to remove the lung lesion of the left lower lobe.

The tumor size was 7 × 6 × 6 cm. It invaded the chest wall, and the intraoperative frozen section showed a poorly differentiated malignant tumor with a reproductive system origin. Because the laparoscopic resection was difficult, it was converted to an axillary small incision, left lower lobectomy, and systematic lymph node dissection.

**Figure 2:**
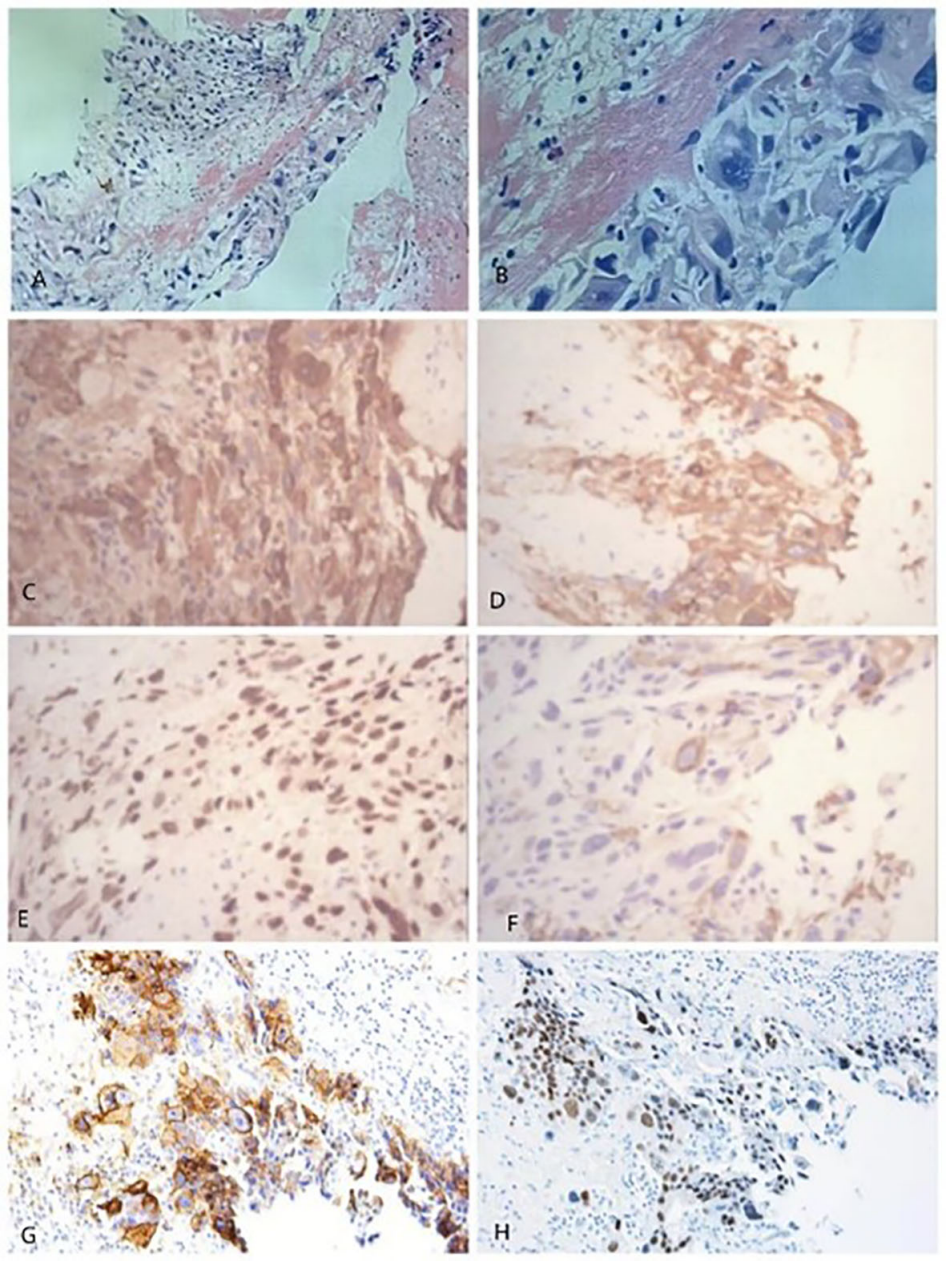
HE staining: Figure **(B)** is a partial enlargement of figure **(A)**. **(B)**: Fibrinous-like material is present in the upper left corner, and neoplastic trophoblasts are present in the lower right corner, with large hyperchromatic nuclei and no villi, as well as strongly positive immunohistochemical staining of the tumor at 40× magnification: **(C)** β-hCG; **(D)** CK 8/18; **(E)** Ki-67; and **(F)** HPL; and at 20× magnification: **(G)** CD10; **(H)** GATA-3. The pathological diagnosis was chorionic epithelial carcinoma of the left lower lobe of the lung, and the diameter of the lesion was approximately 10 cm. Most of the lesion was necrotic, and only a few tumor cells were visible.

HE staining (**Figure 2**) showed a small number of tumor cells. Immunohistochemical examination of the diseased tissues of the patients revealed that tumor cells were strongly positive for CK 8/18, CD10, β-hCG, proliferating cell nuclear antigen Ki-67, human placental lactogen (HPL), and GATA-3 and were negative for P63 and P40. The patient was diagnosed with PPC based on the results of laboratory and pathological examinations.

The patient’s postoperative vital signs were normal, and no serious adverse reactions occurred. We advised the patient to review HCG and to undergo chemotherapy, but the patient refused due to economic and social factors. After a period of postoperative treatment, the patient recovered well and was discharged after completing a series of examinations, and it was recommended that the patient be reexamined regularly. A follow-up 5 years later showed that the patient had recovered well, with no recurrence or metastasis of the lung or other sites detected by chest CT and physical examinations.

## Discussion

**Figure 3:**
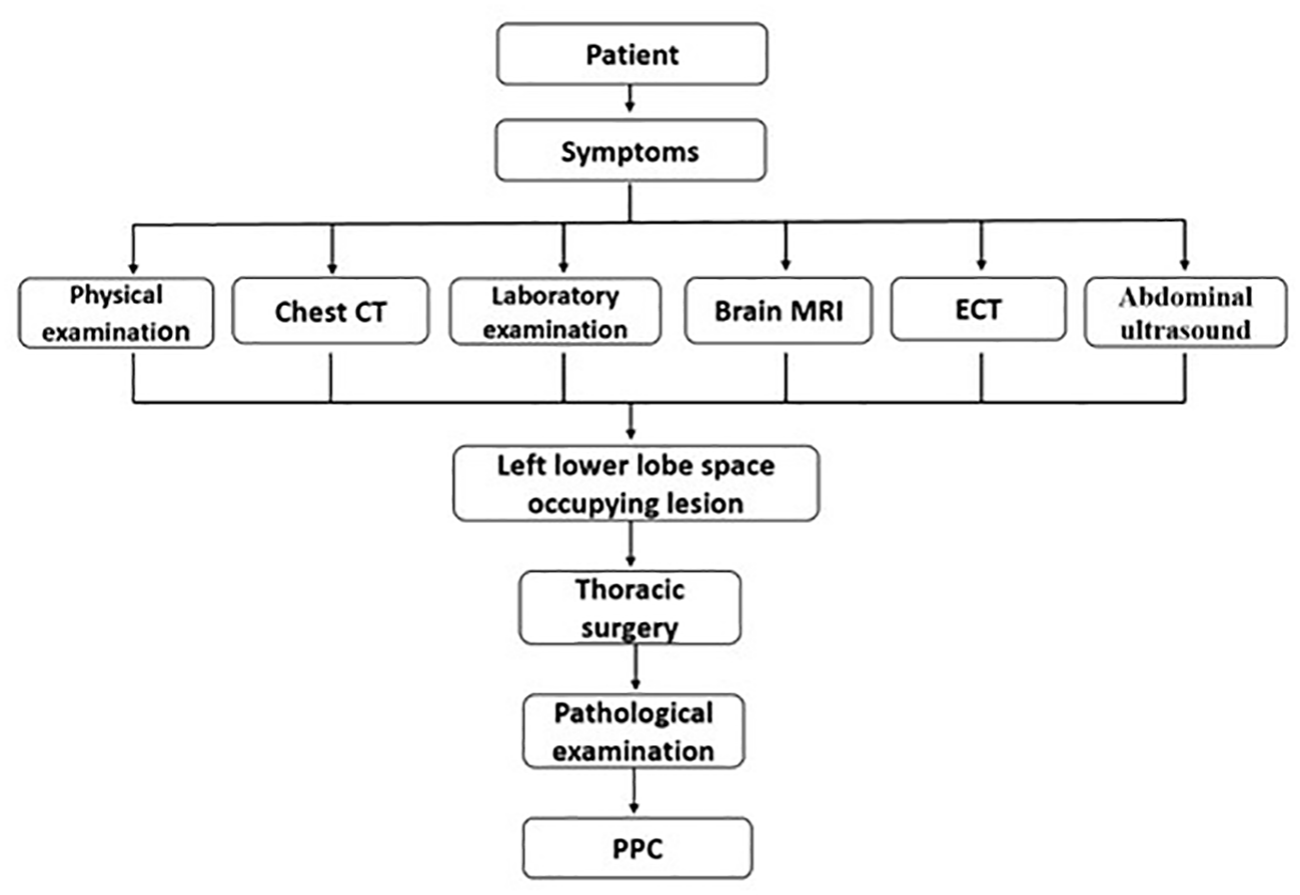
The process of the patient’s visit.

Our report depicts a difficult situation in which the diagnosis of PPC was unexpected in women who were not pregnant but had elevated β-hCG levels (**Figure 3**). As can be seen in this case, no specific features contributed to the diagnosis of PPC, which was confirmed by postsurgical biopsy. Combined with other published cases of primary pulmonary choriocarcinoma (**Table 1**), the preoperative diagnosis of PPC is difficult unless β-hCG is determined for other reasons (12). In this case, the patient’s two CT examinations failed to diagnose the type of disease, demonstrating that CT examinations with high clinical diagnostic value for other common lung diseases are not helpful for the diagnosis in PPC. CT scans, on the other hand, can be used for the precise localization of lesions, as well as for searching for primary and metastatic lesions of tumors due to their convenience and high-density resolution.

**Table 1:** List of primary pulmonary choriocarcinoma cases in recent 3 years.

	Author year	Age, y	Sex	Symptoms	Tumor locations	Therapy
**1.**	Dlewati et al. (3), 2022	54	Female	Dyspnea cough, hemoptysis	Superior lobe of the right lung	Chemotherapy, resection
**2.**	Zhang et al. (7), 2022	65	Male	None	Superior lobe of the left lung	Resection
**3.**	Wu et al. (2), 2020	37	Female	Cough, dyspnea, chest pain, hemoptysis, weight loss	Inferior lobe of the right lung	Resection ×2, chemotherapy
**4.**	Johnson et al. (8), 2022	37	Female	Recurrent syncopal episodes	Superior lobe of the left lung	Resection, chemotherapy
**5.**	Nguyen et al. (9), 2020	42	Male	Chest pain, weight loss	Multiple lesions in both lungs	Resection, chemotherapy
**6.**	Onishi et al. (10), 2021	40	Female	None	Inferior lobe of the right lung	Resection
**7.**	Kim et al. (11), 2020	44	Female	Chest pain, fever	Multiple lesions in both lungs	Chemotherapy

Primary choriocarcinoma, also known as non-gestational choriocarcinoma, is different from gestational choriocarcinoma. The former is not related to pregnancy, originates from primordial germ cells, and can occur in both men and women. It generally occurs in the gonadal organs but also in the midline outside the gonads (such as brain-pineal gland, mediastinum, retroperitoneum), and even other organs (e.g., stomach, lung, pancreas) (7).

It is important to note that primary choriocarcinoma of the lung should be differentiated from giant cell carcinoma of the lung (13), as both diseases are associated with a lung mass and elevated β-hCG levels (11). However, the number and volume of multinucleated giant cells in giant cell carcinoma of the lung are lower than in PPC, and the level of β-hCG elevation is similarly lower. Furthermore, PPC must be differentiated from other types of pulmonary lesions (14). In this case, the patient exhibited a cough, sputum, fever, and other obvious symptoms, which are common manifestations of pulmonary infections, and this is why the patient was initially misdiagnosed with a pulmonary cyst complicated with infection. Pulmonary inflammatory pseudotumor (15) and organizing pneumonia (16) are also symptoms of pulmonary infection that must be differentiated during the diagnosis process. On CT images, organizing pneumonia presents as diffuse lesions in the lungs, whereas pulmonary inflammatory pseudotumors and PPCs appear as nodules or masses in the lungs. PPC, on the other hand, always has an unclear border and may be accompanied by villous bulges. When distinguishing between them is challenging, an enhanced CT (17) or lung biopsy should be performed to further clarify the nature of the disease.

The tumor in this case originated in the lung, which is unusual compared with the previously common choriocarcinoma (18). Although this is not the first case, the conception of the cellular origin and pathogenesis of PPC is still limited (19). Here are several popular theories. The first theory is that it originates from the ectopic position of primordial germ cells in the lungs during embryonic development. The second theory is that PPC is a high-level transformation of non-trophoblastic lung tumors. The third theory is that primary choriocarcinoma of the lung is the same as giant cell carcinoma of the lung (6, 20). Because the patient was not pregnant, the second theory is more likely to explain this occurrence.

Regarding the diagnosis of primary choriocarcinoma, there was no significant difference in the pathological morphology between gestational choriocarcinoma and non-gestational choriocarcinoma, and the immunohistochemical results could be CK (+) and HCG (+), but from the point of origin and tissue, they were significantly different. Gestational choriocarcinoma is a tumor transformed from allogeneic cells with male components, so it has strong immunogenicity or antigenicity, whereas non-gestational choriocarcinoma is a tumor transformed from autologous cells, which has low immunogenicity or antigenicity. The precise differentiation requires polymorphism analysis of the tumor genome. Therefore, it is necessary to check the presence of paternal gene in the tumor tissue to confirm the diagnosis of this patient (21). Short tandem repeat (STR) genotyping is currently the most suitable method for identity detection (22).

Due to the poor survival rate of patients with PPC, early accurate diagnosis and aggressive treatment are crucial. In this case, the follow-up after 5 years showed a good recovery, which again confirmed that the diagnosis was consistent with PPC. Previous studies have shown that patients who are actively treated have longer survival than those who are not treated, and the survival rate of the surgical group is higher than that of the non-surgical group according to the different treatment methods (7). Although PPC is a highly malignant tumor, there is no standard treatment for it due to its rarity. Treatment strategies for PPC in published cases have included complete excision, chemotherapy, radiation therapy, and supportive care, which are the same as those for gestational choriocarcinoma (3, 23). Surgical resection is an effective treatment for PPC, particularly for isolated lung lesions, when combined with postoperative chemotherapy to prevent tumor metastasis to other sites (5, 24). Etoposide, carboplatin, bleomycin, and methotrexate are examples of common chemotherapy drugs (3, 6). Furthermore, patients with choriocarcinoma who are resistant to chemotherapeutic drugs can be treated with a combination of toripalizumab and surgery (25).

In conclusion, primary pulmonary choriocarcinoma is very rare and difficult to diagnose, and elevated β-hCG levels are characteristic of the disease. According to current research, the combination of surgery and chemotherapy has a superior effect. Further research on appropriate treatment options and factors affecting survival is needed to improve the prognosis of patients with PPC.

## Data availability statement

The original contributions presented in the study are included in the article/supplementary material. Further inquiries can be directed to the corresponding author.

## Ethics statement

The studies involving human participants were reviewed and approved by the Institutional Review Board of Shandong Provincial Hospital Affiliated to Shandong First Medical University Subcommittee on Human Studies. The patient provided written informed consent to participate in this study. Written informed consent was obtained from the individual for the publication of any potentially identifiable images or data included in this article.

## Author contributions

NW and NZ wrote the main manuscript text and contributed equally. XZ and YW prepared figures. YF collected the clinical data and imaging data. LG, CL, and MY revised the manuscript text. All authors revised the manuscript and approved the submitted version.

## References

[1] Ben Abdallah I Mlika M Berrazega Y Mejri N Rachdi H El Benna H. Amenorrhea and elevated betahuman chorionic gonadotropin of unknown origin: an unexpected location of choriocarcinoma. Gynecol Oncol Rep (2021) 36:100746. doi: 10.1016/j.gore.2021.100746 33889701 PMC8047451

[2] Wu PS. Primary choriocarcinoma of the lung: a case report and literature review. Int J Clin Exp Pathol (2020) 13(9):2352–5. PMC7539882 33042342

[3] Dlewati MM Gonzalez T Razi SS Hussain SF Bennett J. Primary pulmonary choriocarcinoma treated with neoadjuvant chemotherapy and lobectomy: a case report. Cureus (2022) 14(2):e21931. doi: 10.7759/cureus.21931 35273872 PMC8901110

[4] Ji YS Park SH. Clinical experience of Male primary choriocarcinoma at the Samsung medical center. Cancer Res Treat (2021) 53(3):874–80. doi: 10.4143/crt.2020.1066 PMC8291190 33285049

[5] Chen F Tatsumi A Numoto S. Combined choriocarcinoma and adenocarcinoma of the lung occurring in a man: case report and review of the literature. Cancer (2001) 91(1):123–9. doi: 10.1002/1097-0142(20010101)91:1<123::AID-CNCR16>3.0.CO;2-3 11148568

[6] Rali P Xie J Rali G Rali M Silverman J Malik K. A rare case of metastatic choriocarcinoma of lung origin. Case Rep Pulmonol (2017) 2017:4649813. doi: 10.1155/2017/4649813 29362683 PMC5738576

[7] Zhang X Ding B Chen L Huang X Zhang K Wang Z. Primary pulmonary choriocarcinoma in male: report a case with genetic testing and review of the literature. Transl Cancer Res (2022) 11(6):1844–9. doi: 10.21037/tcr-21-2627 PMC9273666 35836509

[8] Johnson AM Johnson CM Khalil Z Stitzel M Teoh D. Case report: treatment of primary pulmonary choriocarcinoma with lung lobectomy and adjuvant chemotherapy. Gynecol Oncol Rep (2022) 43:101064. doi: 10.1016/j.gore.2022.101064 36092979 PMC9460167

[9] Nguyen HTT Hoang HH Le ATV. A case report of primary pulmonary choriocarcinoma in a man: successful combination of surgery and chemotherapy. Case Rep Oncol (2020) 13(2):923–8. doi: 10.1159/000508744 PMC7443671 32884541

[10] Onishi I Kirimura S Wakejima R Okubo K Odai T Kakuta R. Primary pulmonary choriocarcinoma with a genomic sequence. Pathol Int (2022) 72(2):141–3. doi: 10.1111/pin.13193 34904768

[11] Ikura Y Inoue T Tsukuda H Yamamoto T Ueda M Kobayashi Y. Primary choriocarcinoma and human chorionic gonadotrophin-producing giant cell carcinoma of the lung: are they independent entities? Histopathology (2000) 36(1):17–25. doi: 10.1046/j.1365-2559.2000.00789.x 10632747

[12] Kamata S Sakurada A Sato N Noda M Okada Y. A case of primary pulmonary choriocarcinoma successfully treated by surgery. Gen Thorac Cardiovasc Surg (2017) 65(6):361–4. doi: 10.1007/s11748-016-0666-8 27236469

[13] Habib S Leifer LE Azam M Siddiqui AH Rajdev K Chalhoub M. Giant cell carcinoma of the lung successfully treated with surgical resection and adjuvant vinorelbine and cisplatin. Respir Med Case Rep (2018) 25:300–2. doi: 10.1016/j.rmcr.2018.10.012 PMC6199182 30370215

[14] Kim JH Cha MJ Kim MK Chung YJ Lee EJ. Disseminated primary pulmonary choriocarcinoma successfully treated by chemotherapy: a case report and literature review. Cancer Invest (2020) 38(8-9):493–501. doi: 10.1080/07357907.2020.1804575 32845165

[15] Kim JH Cho JH Park MS Chung JH Lee JG Kim YS. Pulmonary inflammatory pseudotumor–a report of 28 cases. Korean J Intern Med (2002) 17(4):252–8. doi: 10.3904/kjim.2002.17.4.252 PMC4531693 12647641

[16] Cherian SV Patel D Machnicki S Naidich D Stover D Travis WD. Algorithmic approach to the diagnosis of organizing pneumonia: a correlation of clinical, radiologic, and pathologic features. Chest (2022) 162(1):156–78. doi: 10.1016/j.chest.2021.12.659 PMC9899643 35038455

[17] Wang XL Shan W. Application of dynamic CT to identify lung cancer, pulmonary tuberculosis, and pulmonary inflammatory pseudotumor. Eur Rev Med Pharmacol Sci (2017) 21(21):4804–9. 29164583

[18] Jiao L Ghorani E Sebire NJ Seckl MJ. Intraplacental choriocarcinoma: systematic review and management guidance. Gynecol Oncol (2016) 141(3):624–31. doi: 10.1016/j.ygyno.2016.03.026 27020699

[19] Takahashi T Kobayashi R. Choriocarcinoma syndrome after resection of primary pulmonary choriocarcinoma: report of a case. Surg Case Rep (2016) 2(1):122. doi: 10.1186/s40792-016-0227-5 27807803 PMC5093096

[20] Słodkowska J Kamiński Z Decker E Broniek A Radomski P. Are lung choriocarcinoma and giant cell carcinoma producing chorionic gonadotropins just variants of the same neoplasm? Pneumonol Alergol Pol (1996) 64(11-12):798–804. 9162326

[21] Jia N Chen Y Tao X Ou E Lu X Feng W. A gestational choriocarcinoma of the ovary diagnosed by DNA polymorphic analysis: a case report and systematic review of the literature. J Ovarian Res (2017) 10(1):46. doi: 10.1186/s13048-017-0334-3 28728581 PMC5520233

[22] da Silva FF Barata R Rolim I Carvalheiro C Gil N Pantarotto M. Case report: using DNA short tandem repeats to confirm nongestational origin of pulmonary choriocarcinoma. Front Oncol (2022) 12:1001627. doi: 10.3389/fonc.2022.1001627 36324567 PMC9619092

[23] Yun J Lee SW Lim SH Kim SH Kim CK Park SK. Successful treatment of a high-risk nonseminomatous germ cell tumor using etoposide, methotrexate, actinomycin d, cyclophosphamide, and vincristine: a case report. World J Clin Cases (2020) 8(21):5334–40. doi: 10.12998/wjcc.v8.i21.5334 PMC7674725 33269267

[24] Ngu SF Ngan HYS. Surgery including fertility-sparing treatment of GTD. Best Pract Res Clin Obstet Gynaecol (2021) 74:97–108. doi: 10.1016/j.bpobgyn.2020.10.005 33127305 PMC7547826

[25] Liu X Li X Qu H Zhang S Zhang R Du Z. Effectiveness and safety of toripalimab combination therapies for patients with chemo-resistant choriocarcinoma. Front Oncol (2022) 12:815917. doi: 10.3389/fonc.2022.815917 35494052 PMC9047865

